# Exploring the Cardioprotective Spectrum–Effect Relationship of *Apocynum venetum* L. Using a Zebrafish Model

**DOI:** 10.3390/ph19060879

**Published:** 2026-05-31

**Authors:** Wenli Xie, Xinhai Cui, Yaobo Zhang, Yuhan Zhai, Xianjun Fu, Kuo Xu

**Affiliations:** 1 Research Institute of Marine Traditional Chinese Medicine, The SATCM’s Key Unit of Discovering and Developing New Marine TCM Drugs, Key Laboratory of Marine Traditional Chinese Medicine in Shandong Universities, Shandong University of Traditional Chinese Medicine, Jinan 250355, China; 2 Qingdao Academy of Chinese Medical Sciences Shandong University of Traditional Chinese Medicine, Qingdao Key Laboratory of Research in Marine Traditional Chinese Medicine, Qingdao Key Technology Innovation Center of Marine Traditional Chinese Medicine’s Deep Development and Industrialization, Qingdao 266114, China; 3 College of Traditional Chinese Medicine, Shandong University of Traditional Chinese Medicine, Jinan 250355, China; 4 National Center of Technology Innovation for Comprehensive Utilization of Saline-Alkali Land, Dongying 257345, China

**Keywords:** *Apocynum venetum* L., zebrafish, cardioprotective activity, chlorogenic acid, spectrum–effect relationship

## Abstract

**Background/Objectives:** *Apocynum venetum* L. leaves have long been used in traditional medicine and have shown cardioprotective potential, but the active constituents responsible for these effects remain unclear. This study aimed to identify cardioprotective constituents in the n-butanol fraction of *A. venetum* leaves and clarify their mechanisms through chemical profiling, zebrafish phenotyping, and cell-based validation. **Methods**: The n-butanol extract was fractionated into S1–S6 and characterized by HPLC and UPLC–QE–Orbitrap–MS/MS. Cardioprotective effects were assessed in zebrafish myocardial injury models. Spectrum–effect relationships were analyzed using grey relational analysis (GRA) and orthogonal partial least squares (OPLS), and key compounds were validated in zebrafish. Transcriptomic analysis, qRT–PCR, molecular docking, and isoprenaline (ISO)-induced H9c2 cell assays were used to explore mechanisms and synergistic effects. **Results**: Fingerprinting analysis of subfractions S1–S6 revealed 12 common peaks and ten compounds. S1–S4 showed stronger protection against zebrafish myocardial injury than S5–S6. Spectrum–effect analysis indicated that chlorogenic acid (CGA) and 4-caffeoylquinic acid (4-CQA) were the major activity-associated constituents. CGA produced the most pronounced cardioprotective effect in zebrafish and was associated with PPARα-related metabolic pathways. In H9c2 cells, CGA combined with myricetin 3-*O*-glucoside showed synergistic protection. Quantitative analysis showed that CGA and 4-CQA were predominantly distributed in S1–S3, consistent with the stronger activity of these fractions. **Conclusions**: This study applied a spectrum–effect relationship strategy to identify CGA and 4-CQA as major cardioprotective constituents in the n-butanol fraction of *A. venetum* leaves, supporting the potential synergistic contribution of CGA-dominated constituent combinations and providing a basis for further pharmacological investigation.

## 1. Introduction

*Apocynum venetum* L. is a perennial herbaceous species of the Apocynaceae family that is widely distributed in the arid and saline–alkali regions of China, including northwestern areas such as Xinjiang and coastal provinces such as Shandong [1]. It has been consistently documented in the Chinese Pharmacopoeia since 1977 and is clinically utilized for the treatment of hypertension [2]. Previous phytochemical studies have shown that the leaves of *A. venetum* are rich in flavonoids, phenolic acids, and phenylpropanoids, which are considered the primary material basis for its biological activities [3,4]. In addition to its traditional medicinal use, increasing evidence has demonstrated the cardioprotective potential of *A. venetum* leaves. Multiple experimental findings indicate that it effectively mitigates myocardial injury and improves cardiac function through various mechanisms, including enhancing mitochondrial function, reducing myocardial apoptosis, and regulating gut microbiota metabolism [5,6,7,8,9,10].

However, the chemical composition of *A. venetum* leaves is highly complex, and the distribution of constituents differs substantially among fractions with different polarities. Most previous studies have focused primarily on crude extracts, making it difficult to identify the specific compounds responsible for the cardioprotective effects [5,6,7,8,9,10]. In addition, conventional chromatographic fingerprinting approaches have been used mainly for qualitative or semi-quantitative characterization of chemical constituents, frequently lacking direct correlation with biological efficacy. The systematic integration of chemical profiling with pharmacological evaluation for active constituent discovery remains necessary [11].

Our previous study showed that the n-butanol fraction of *A. venetum* leaves exerted stronger cardioprotective effects against drug-induced myocardial injury in zebrafish than the total extract, suggesting that this fraction may be enriched in bioactive constituents [12]. To clarify the material basis underlying its efficacy, this study established the HPLC fingerprint of *A. venetum* leaves and evaluated the spectrum–effect relationship between their chemical profiles and the protective effects against drug-induced myocardial injury using grey relational analysis (GRA) and orthogonal partial least squares (OPLS). By integrating bioactivity validation in zebrafish and H9c2 cell models with transcriptomics, molecular docking, and quantitative component analysis, the key bioactive constituents of *A. venetum* leaves were screened and identified. This integrated spectrum–effect strategy provides a practical reference for screening bioactive constituents and further exploring the pharmacological basis and preclinical relevance of *A. venetum*.

## 2. Results

### 2.1. HPLC Fingerprint Analysis and Component Identification

**Figure 1:**
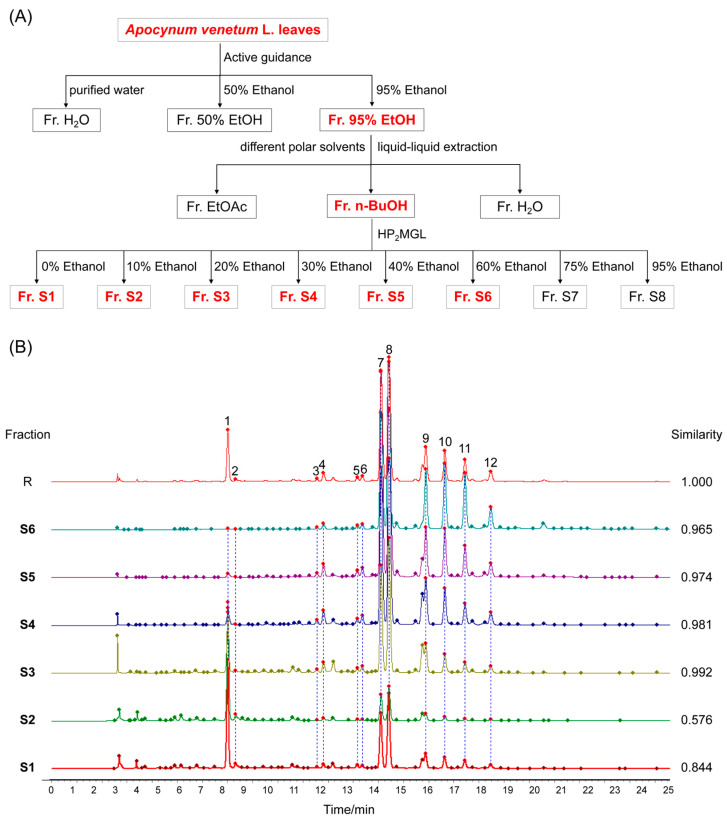
Preparation and chromatographic characterization of subfractions S1–S6 from *A. venetum* leaves. (**A**) Activity-guided fractionation process of *A. venetum* leaves; (**B**) similarity and common peaks of the HPLC fingerprints of subfractions S1–S6 of *A. venetum* leaves.

The n-BuOH fraction was subjected to HP_2_MGL macroporous adsorption resin column chromatography and eluted with different ethanol concentrations, yielding a total of eight fractions (S1–S8). The preparation process is illustrated in Figure 1A. As the final freeze-dried subfractions used for subsequent chemical analysis and bioactivity evaluation, the dry powder weights for each fraction were as follows: S1 (12.91 g), S2 (5.89 g), S3 (6.87 g), S4 (8.39 g), S5 (9.46 g), S6 (2.41 g), S7 (0.72 g), and S8 (0.39 g). Owing to the limited available amounts of S7 and S8 and preliminary toxicity tests indicating their higher toxicity, these two subfractions were excluded from subsequent activity experiments.

The HPLC fingerprint profiles of subfractions S1–S6 are shown in Figure 1B. HPLC analysis identified a total of 12 characteristic peaks with good separation and resolution. As shown in Figure 1B, the types of major components were largely consistent across samples, although their contents and proportions exhibited certain variations. Similarity analysis revealed similarity values of 0.844 and 0.576 for samples S1 and S2, respectively, whereas all the other samples exhibited similarity values exceeding 0.95. Sample S2 demonstrated the lowest similarity value, indicating significant differences in chemical composition compared to other regions. Method validation results further confirmed the method’s excellent precision, stability, and repeatability (Supplementary Material, Result S1). In particular, the lower similarity values of S1 and S2 mainly resulted from noticeable differences in several major peaks, suggesting variations in the relative abundance of key compounds.

The obtained mass spectrometry data were systematically analyzed using Thermo Xcalibur 4.7 software. Structural identification and characterization of the detected constituents were accomplished by integrating accurate mass measurements, characteristic MS/MS fragment ions, relative retention behavior, and reference standard comparison where available. As summarized in Table 1, ten peaks were structurally characterized, including P1 (CGA), P2 (4-CQA), P3 (myricetin 3-*O*-galactoside, Myr-3-Gal), P4 (myricetin 3-*O*-glucoside, Myr-3-Glc), P7 (hyperoside), P8 (isoquercitrin), P9 [quercetin 3-*O*-(6″-*O*-malonyl)-galactoside], P10 (kaempferol 3-*O*-galactoside), P11 (apigenin 7-*O*-glucoside), and P12 [quercetin 3-*O*-(6″-*O*-acetyl)-glucoside], whereas P5 and P6 remained unidentified under the current experimental conditions because of their extremely low abundance. Therefore, these two peaks should presently be regarded as bioactivity-associated chromatographic signals rather than definitively identified active constituents. The total ion chromatograms in both positive and negative ion modes, along with the MS/MS fragmentation patterns and proposed cleavage pathways of compounds corresponding to peaks 1–4, are presented in Supplementary Material, Result S2, highlighting key fragment ions and inferred structural breakpoints.

### 2.2. Protective Effects of Subfractions S1–S6 on Myocardial Injury in Zebrafish

#### 2.2.1. Effect on Myocardial Injury Phenotypes and Blood Flow

**Figure 2:**
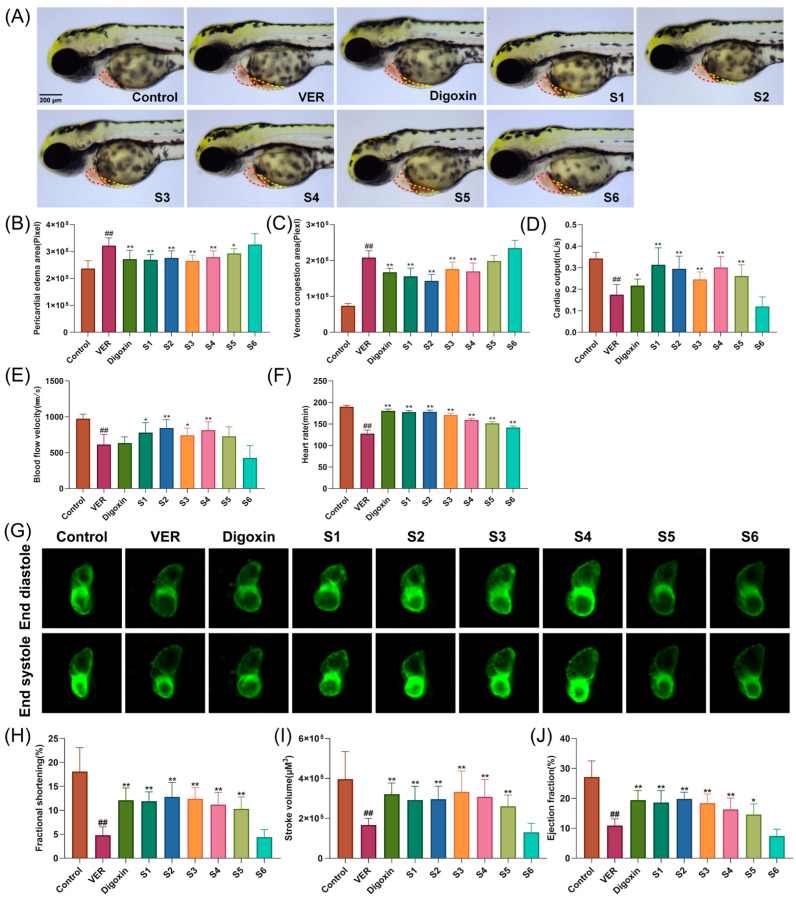
Evaluation of the efficacy of subfractions S1–S6 of Fr. n-BuOH in improving the levels of myocardial injury indicators in zebrafish (n = 10). (**A**) Comparative images of cardiac function improvement in wild-type AB zebrafish (×50); the red dashed area indicates the PA, and the yellow dashed area indicates the VA; (**B**) PA; (**C**) VA; (**D**) CO; (**E**) BFV; (**F**) HR; (**G**) comparative images of cardiac function improvement in Tg(*myl7:GFP*) zebrafish (×11.2); (**H**) FS; (**I**) SV; (**J**) EF; ^##^ *p* < 0.01 vs. control group; ** *p* < 0.01, * *p* < 0.05 vs. model group.

As shown in Figure 2, in wild-type AB zebrafish, the model group exhibited a typical myocardial injury phenotype compared to the control group. This phenotype was primarily manifested by significant increases in pericardial edema area (PA; red dashed area) and venous congestion area (VA; yellow dashed area) (Figure 2A–C), accompanied by significant decreases in cardiac output (CO), blood flow velocity (BFV), and heart rate (HR) (Figure 2D–F), indicating the successful establishment of the VER-induced zebrafish myocardial injury model. Pharmacodynamic comparison revealed marked differences among subfractions S1–S6 in ameliorating myocardial injury. Specifically, S1–S4 significantly reduced PA and VA while restoring CO, BFV, and HR levels, with overall efficacy comparable to that of the digoxin-positive control group. In contrast, S5–S6 demonstrated inferior effects.

#### 2.2.2. Effect on Cardiac Function

In Tg(*myl7:GFP*) transgenic zebrafish, the model group exhibited significantly lower fractional shortening (FS), stroke volume (SV), and ejection fraction (EF) than the control group (Figure 2G–J), confirming the successful establishment of the verapamil (VER)-induced myocardial injury model. Pharmacodynamic comparison revealed that fractions S1–S5 effectively mitigated decreases in cardiac function indicators, demonstrating promising cardioprotective potential. Conversely, fraction S6 did not significantly improve across functional parameters. These findings align with the morphological observations in Section 2.2.1 and further suggest a graded distribution of cardioprotective activity among subfractions S1–S6, providing a basis for subsequent active compound screening.

### 2.3. Spectrum–Effect Relationship Between the HPLC Fingerprint and Anti-Myocardial Injury Activity

#### 2.3.1. Analysis Results Based on the GRA Method

**Figure 3:**
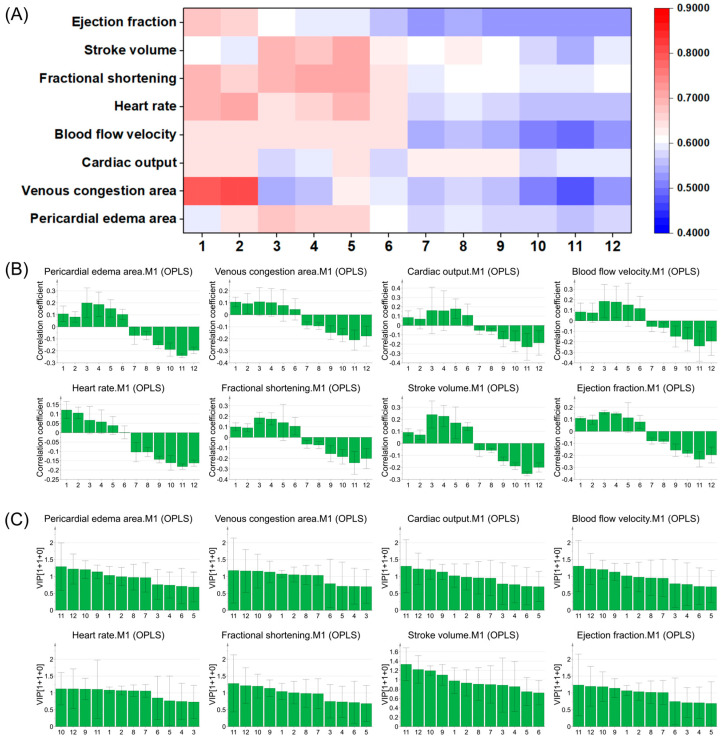
Spectrum–effect relationship analysis based on GRA and OPLS. (**A**) GRA-based heatmap of the spectrum–effect relationship; (**B**) OPLS correlation coefficient of the myocardial injury evaluation index (PA, VA, CO, BFV, HR, FS, SV and EF); (**C**) VIP values of the myocardial injury evaluation indicators (PA, VA, CO, BFV, HR, FS, SV and EF).

The spectrum–effect correlation model constructed on the basis of GRA revealed associations between 12 common peaks across subfractions S1–S6 and 8 activity indicators. The results revealed that the grey correlation coefficients between these 12 common peaks and each indicator ranged from 0.4679 to 0.8044 (Figure 3A). Further comparison revealed that each common peak exhibited cross-influences across multiple indicators. In the correlation ranking, the average grey correlation degrees of P1, P2, P5, P4, P3, and P6 all exceeded 0.6, whereas the remaining peaks were below 0.6. Generally, a grey correlation degree above 0.6 indicates a correlation between a component and pharmacological efficacy, whereas a value above 0.8 signifies a significant correlation. Therefore, chromatographic peaks 1–6 are closely associated with the anti-myocardial injury activity of *A. venetum* leaves and may represent its key bioactive components.

#### 2.3.2. Analysis Results Based on the OPLS Method

The results of the OPLS analysis are shown in Figure 3B,C. The OPLS models were established separately for the eight myocardial injury-related indicators, with R^2^(cum) values ranging from 0.804 to 0.998 and Q^2^(cum) values ranging from 0.641 to 0.996, indicating acceptable fitting and predictive performance. Detailed model parameters are provided in Supplementary Materials, Table S3. In the regression model, the absolute value of the correlation coefficient reflects the strength of influence of each chromatographic peak on the activity indicators, whereas the sign (positive or negative) indicates a positive or inverse relationship with efficacy. Overall, peaks 1–6 were positively associated with cardioprotective efficacy, whereas peaks 7–12 showed negative associations. The VIP value is a key metric for evaluating the explanatory contribution of variables, and variables with VIP > 1 are generally considered important contributors to the model. Comprehensive analysis revealed that both peak 1 (CGA) and peak 2 (4-CQA) had VIP values > 1, indicating their strong role in improving myocardial injury. Although peaks 3–6 had VIP values < 1, they still showed positive associations with cardiac function-related indicators. Combined with the GRA results, these findings connected the 12 predicted compounds with their cardioprotective bioactivities: P1 (CGA) and P2 (4-CQA) were identified as the major bioactivity-associated compounds, P3–P6 were positively associated with the activity and may contribute as auxiliary constituents, whereas P7–P12 showed weaker or negative associations, suggesting relatively limited direct contributions to the observed cardioprotective effects under the present experimental conditions. These results suggest that the overall cardioprotective effect may arise from the combined action of major and auxiliary activity-associated compounds rather than from a single compound alone.

### 2.4. Protective Effects of Key Components Against Myocardial Injury in Zebrafish

**Figure 4:**
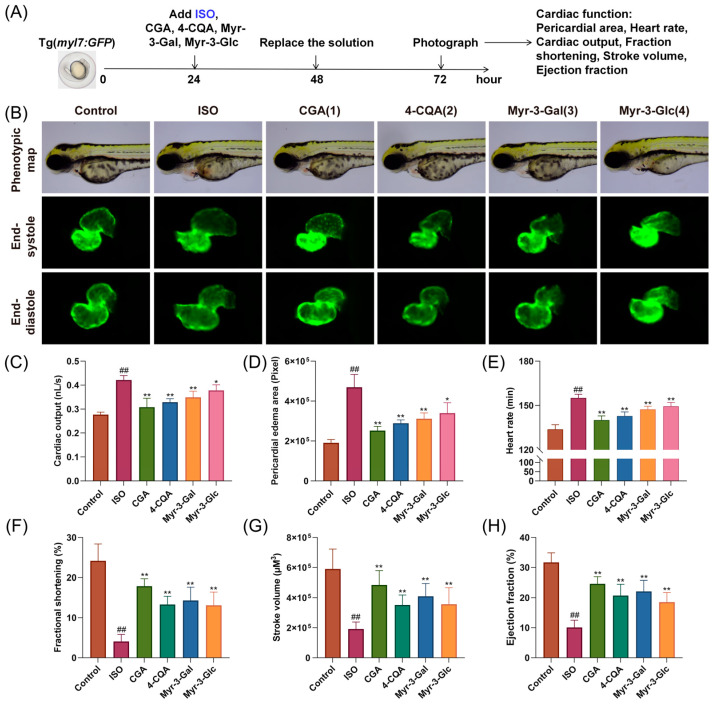
Evaluation of the cardioprotective effects of key active components from *A. venetum* leaves on myocardial injury-related indicators in zebrafish (n = 10). (**A**) Drug administration scheme for the ISO-induced myocardial injury zebrafish model; (**B**) representative bright-field (×50) and fluorescent (×11.2) images of improved cardiac function phenotypes in zebrafish; (**C**) CO; (**D**) PA; (**E**) HR; (**F**) FS; (**G**) SV; (**H**) EF. ^##^ *p* < 0.01 vs. the control group; ** *p* < 0.01, * *p* < 0.05 vs. the model group.

To validate the active compounds identified by the spectral–effect relationship model, peaks P1–P4 were prioritized for further evaluation because their structures were identified and the corresponding reference standards were available. An ISO-induced zebrafish embryo myocardial injury model was employed for this purpose. This model induces cardiac hypertrophy and cardiomyocyte apoptosis, accompanied by ventricular enlargement and reduced ejection fraction, thereby providing a useful zebrafish model for preliminary evaluation of myocardial injury-related phenotypes [13,14,15]. The experimental procedure is shown in Figure 4A. The results revealed a pronounced cardiac hypertrophy phenotype in the model group of embryos, which was further confirmed by fluorescence microscopy, which revealed morphologically abnormal hearts characterized by elongated ventricles (Figure 4B). Quantitative analysis revealed typical pathological alterations in the model group, such as a significantly increased pericardial edema area (+146.0%), elevated heart rate (+15.9%), and increased cardiac output (+52.3%). This was accompanied by a decreased fractional shortening (−83.1%), reduced stroke volume (−67.6%), and decreased ejection fraction (−68.1%), indicating impaired ventricular filling and contractile dysfunction. Notably, treatment with chromatographic peaks 1–4 significantly alleviated ISO-induced cardiac morphological abnormalities, effectively maintained cardiac structural integrity, and restored relevant functional indicators to near-normal levels to varying degrees (Figure 4C–H). On the basis of the comprehensive improvement across all the indicators, CGA demonstrated the most pronounced cardioprotective effects.

### 2.5. Potential Mechanism of the Effects of CGA on Myocardial Injury in Zebrafish

#### 2.5.1. Transcriptome Sequencing Analysis

**Figure 5:**
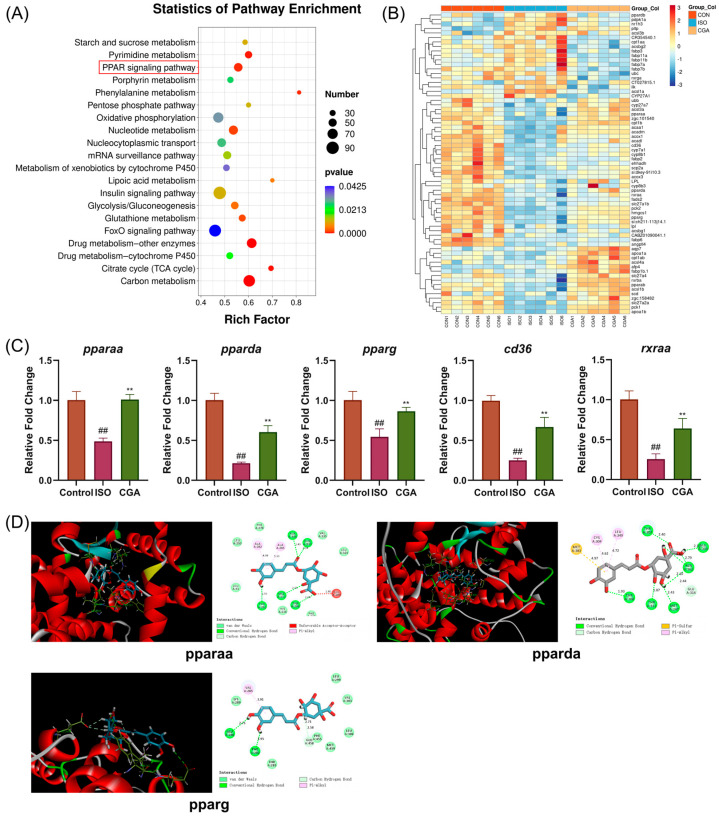
Key signaling pathways regulated by CGA in myocardial injury. (**A**) KEGG pathway enrichment bubble plot; (**B**) heatmap of gene expression in the PPAR signaling pathway; (**C**) regulatory effects of CGA on key PPAR genes (^##^ *p* < 0.01 vs. the control group; ** *p* < 0.01 vs. the model group); (**D**) molecular docking conformations of CGA with PPAR family proteins.

Given that CGA had the most pronounced protective effect in the ISO-induced myocardial injury model in zebrafish, it was selected as a representative active compound for transcriptomic sequencing. Bioinformatic analysis was performed using the OmicStudio tools at https://www.omicstudio.cn/tool (accessed on 24 February 2025). The RNA-seq results revealed 631 upregulated genes and 280 downregulated genes between the control and ISO groups, whereas 573 upregulated genes and 495 downregulated genes were detected between the ISO and CGA groups (Supplementary Material, Result S3). GO annotation indicated that these genes were involved primarily in drug and energy metabolism, redox processes, cytoskeletal functions, and the endoplasmic reticulum (Result S3, Figure S2C). KEGG analysis revealed significant enrichment in multiple metabolic and signaling pathways (Figure 5A). The enriched metabolic pathways mainly included carbon metabolism, glycolysis/gluconeogenesis, the citrate cycle, oxidative phosphorylation, the pentose phosphate pathway, glutathione metabolism, nucleotide/pyrimidine metabolism, and cytochrome P450-related pathways. The enriched signaling pathways included the PPAR signaling pathway, insulin signaling pathway, and FoxO signaling pathway. Notably, the PPAR signaling pathway plays a central role in the pathogenesis of cardiac hypertrophy and heart failure [16]. The enrichment of this pathway indicates that PPAR signaling may be involved in the potential protective effects of CGA against cardiac injury (Figure 5B).

#### 2.5.2. qRT–PCR Analysis

To validate the reliability of the Illumina RNA-seq results and further confirm the expression changes of key genes in the PPAR signaling pathway, *cd36*, *rxraa*, *pparaa*, *pparda* and *pparg* were selected for qRT–PCR analysis in this study. The results (Figure 5C) revealed that compared with those in the control group, the mRNA expression levels of these genes were significantly lower in the ISO model group (*p* < 0.01). Following CGA intervention, the expression levels of these genes increased to varying degrees (*p* < 0.01), with a trend consistent with the RNA-seq results. These findings suggest that CGA potentially regulated the transcription of PPAR pathway-related genes.

#### 2.5.3. Molecular Docking

Molecular docking analysis was performed to simulate the binding patterns of CGA with PPARα (pparaa), PPAR-β/δ (pparda), and PPARγ (pparg) proteins (Figure 5D). The results revealed binding energies of −29.27 kcal/mol, −21.26 kcal/mol, and −28.82 kcal/mol for CGA with pparaa, pparda, and pparg, respectively. Of note, all of these values were negative, indicating strong binding stability with each PPAR protein. Notably, the binding energy of CGA with pparaa was the lowest, suggesting that this interaction was the most robust and stable. Given that PPARα is highly enriched in cardiac tissue and functions as a central regulator of myocardial energy metabolism and cellular homeostasis, this preferential binding supports its biological relevance in the heart [17,18,19]. Therefore, CGA may act as a potential PPARα agonist, alleviating myocardial injury by modulating downstream signaling processes.

### 2.6. Protective Effects of Key Components Against ISO-Induced H9c2 Cell Injury

#### 2.6.1. Effects of CGA, 4-CQA, Myr-3-Gal and Myr-3-Glc

**Figure 6:**
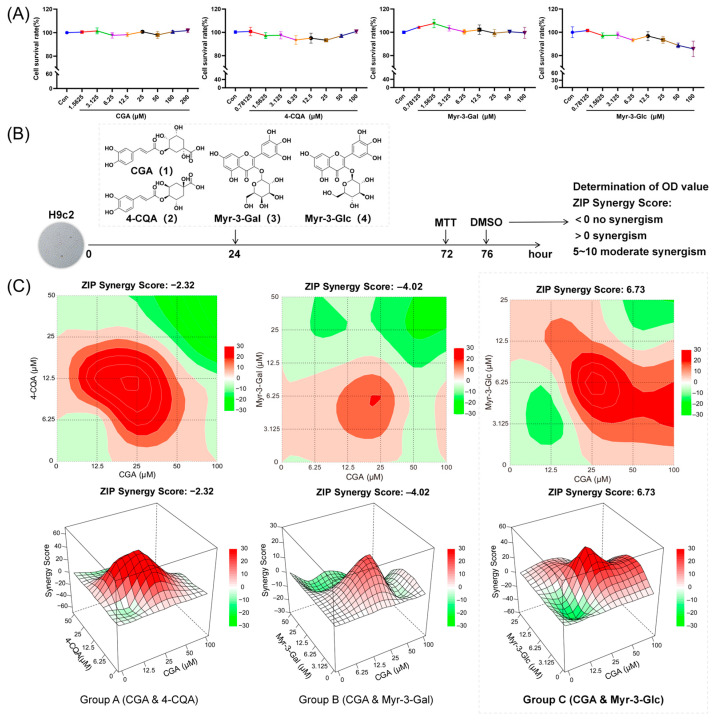
Evaluation of drug synergies. (**A**) Viability of H9c2 cells treated with different drugs (n = 4); (**B**) study design. H9c2 cells were treated with two compounds to assess drug sensitivity. (**C**) The synergistic effects were quantified using the ZIP synergy scores (ZIP synergy scores greater than 0 were considered indicative of synergism).

To validate the candidate compounds identified through spectrum–effect analysis, we evaluated their cytotoxicity in H9c2 cells. MTT assays revealed that CGA (0–200 μM), 4-CQA (0–100 μM), Myr-3-Gal (0–100 μM), and Myr-3-Glc (0–100 μM) did not significantly affect cell viability within the tested concentration ranges (Figure 6A). Because cell viability did not decrease to 50% at the highest tested concentrations, exact cytotoxic IC_50_ values could not be reliably calculated. Therefore, the cytotoxic IC_50_ values were reported as CGA > 200 μM, 4-CQA > 100 μM, Myr-3-Gal > 100 μM, and Myr-3-Glc > 100 μM. After confirming the absence of obvious cytotoxicity, the four candidate compounds were evaluated in combination with ISO in the H9c2 cardiomyocyte injury model at concentrations ranging from 1.5 to 100 μM. Among them, CGA exhibited the most pronounced and consistent protective effects, significantly attenuating ISO-induced cellular damage across the 3.125–100 μM range. In comparison, 4-CQA and Myr-3-Gal conferred moderate protection, with significant effects observed at 12.5–50 μM. Myr-3-Glc exerted relatively weak activity, showing significant protection only at 12.5 μM and 25 μM (Supplementary Material, Result S4).

#### 2.6.2. Synergistic Effects of CGA-Dominated Combinations

To further refine the selection of active ingredient combinations, synergistic effect experiments were conducted as described in Section 2.6.1. Considering that CGA ranked first in both the GRA and OPLS spectral efficacy analyses and demonstrated optimal activity in the chronic myocardial injury model and H9c2 cells, we focused on evaluating the effects of CGA-based dual-drug combinations in H9c2 cells. The specific combinations included Group A (CGA+4-CQA), Group B (CGA+Myr-3-Gal), and Group C (CGA+Myr-3-Glc). Following treatment with different concentrations, cell viability was assessed via the MTT assay, and quantitative evaluation was performed using SynergyFinder+ (https://synergyfinder.org/, accessed on 29 July 2025) to calculate ZIP synergy scores (Figure 6B). A ZIP score > 0 indicates synergistic effects. The results revealed that the combination of CGA+Myr-3-Glc (Group C) achieved a ZIP score of 6.73, indicating significant synergy (Figure 6C).

### 2.7. Mechanism of Action of Group C Against ISO-Induced H9c2 Cell Injury

#### 2.7.1. Regulation of the PPARα/RXRα Signaling Pathway

**Figure 7:**
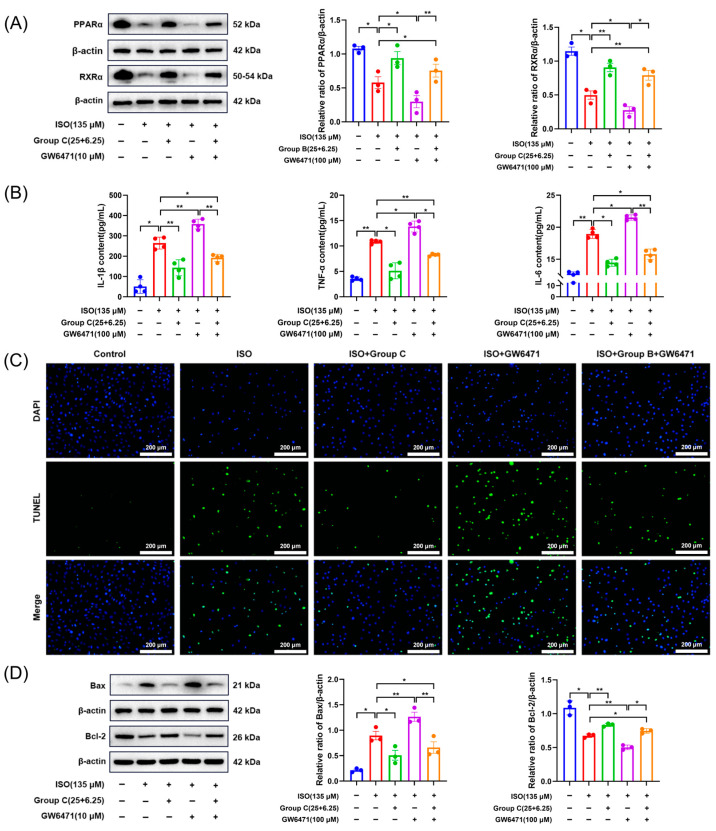
Group C alleviates ISO-induced H9c2 cell damage through the activation of PPARα/RXRα. (**A**) Western blot analysis of PPARα and RXRα expression in H9c2 cells (n = 3). (**B**) Inflammatory cytokine (IL-1β, TNF-α and IL-6) levels in H9c2 cells (n = 4). (**C**) The percentage of TUNEL-positive cells detected by TUNEL staining (n = 3). (**D**) Western blot analysis of Bax and Bcl-2 expression in H9c2 cells (n = 3). ** *p* < 0.01, * *p* < 0.05 between the indicated groups.

Group C contained 25 μM CGA and 6.25 μM Myr-3-Glc, with CGA as the major component. Integration of the results of the transcriptomic and molecular docking analyses indicated that CGA may exert cardioprotective effects through the regulation of PPARα. PPARα activation offers cardioprotective benefits, whereas its inhibition leads to apoptosis and heart dysfunction [20,21,22]. To further clarify whether Group C exerts its anti-myocardial injury effects via activation of the PPARα/RXRα signaling pathway, H9c2 cells were cotreated with CGA and the PPARα inhibitor GW6471. Western blot analysis (Figure 7A) revealed that Group C significantly upregulated PPARα and RXRα protein expression, whereas these effects were markedly attenuated in both the ISO-treated group and the GW6471-treated group.

#### 2.7.2. Modulation of Inflammatory Responses

Previous studies have shown that PPARα agonists markedly suppress inflammatory cytokine expression in cardiomyocytes [23]. In this study, GW6471 treatment markedly increased the expression of inflammatory cytokines (IL-1β, TNF-α, and IL-6), which exceeded the levels observed in the ISO group. Conversely, Group C treatment significantly reduced the expression of these cytokines, effectively counteracting ISO-induced inflammatory activation (Figure 7B). The results indicate that Group C alleviates ISO-induced cellular injury by suppressing myocardial inflammation through the maintenance of PPARα activity.

#### 2.7.3. Inhibition of Cardiomyocyte Apoptosis

**Figure 8:**
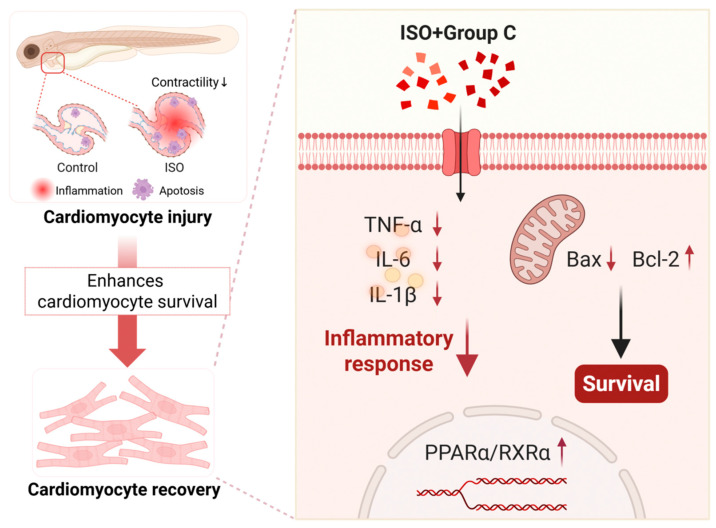
Schematic illustration of the protective mechanism of Group C against ISO-induced H9c2 cell damage. Created in BioRender. Xie, W. (2026) https://BioRender.com/pw6ev4v, accessed on 24 May 2026.

Cardiomyocyte apoptosis was evaluated by TUNEL fluorescence staining (Figure 7C). Compared with the control treatment, ISO treatment significantly increased apoptosis, and GW6471 treatment further exacerbated this effect. Conversely, Group C treatment markedly reduced the number of apoptotic cells compared with both the ISO (*p* < 0.01) and inhibitor groups (*p* < 0.01), indicating a potent anti-apoptotic effect. Western blotting further corroborated these findings (Figure 7D). Compared with that in the control group, Bcl-2 expression in the ISO model group was significantly downregulated (*p* < 0.05), whereas Bax expression was markedly upregulated (*p* < 0.05). These alterations were further exacerbated by GW6471 treatment (*p* < 0.01). Group C cotreatment significantly reduced Bax expression (*p* < 0.05) and increased Bcl-2 expression (*p* < 0.01), further supporting its anti-apoptotic role. Collectively, these findings indicate that PPARα activation is pivotal to the cardioprotective effects of Group C. These effects are achieved through the suppression of inflammatory responses and inhibition of cardiomyocyte apoptosis, ultimately attenuating ISO-induced injury in H9c2 cells (Figure 8).

### 2.8. Quantitative Distribution of Activity-Associated Constituents and Implications for Quality Assessment

**Figure 9:**
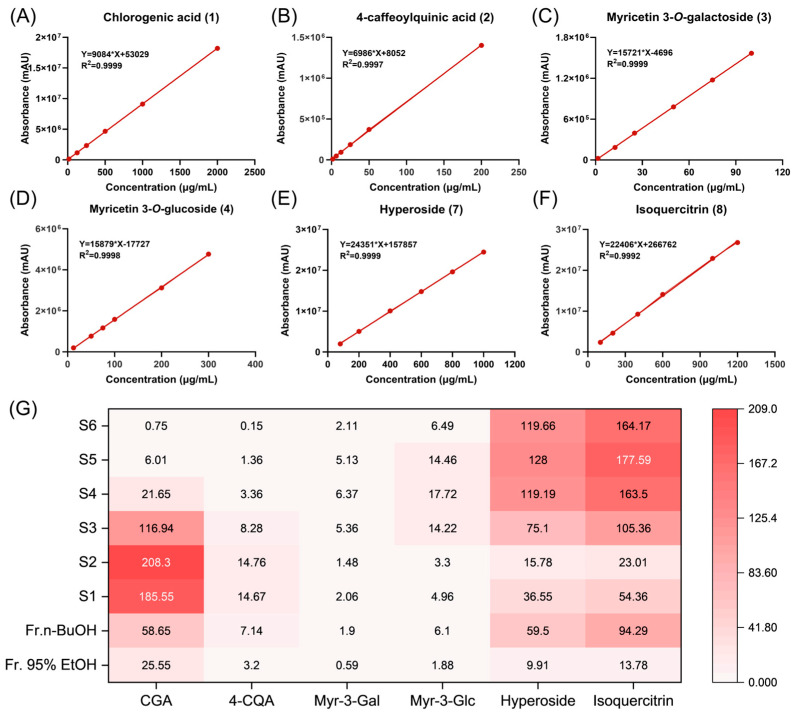
Quantitative distribution of six representative activity-associated constituents in Fr. 95% EtOH, Fr. n-BuOH, and subfractions S1–S6 of *A. venetum* leaves. (**A**–**F**) Calibration curves of CGA (1), 4-CQA (2), Myr-3-Gal (3), Myr-3-Glc (4), hyperoside (7), and isoquercitrin (8). (**G**) Heatmap showing the contents (mg/g) of the six constituents in Fr. 95% EtOH, Fr. n-BuOH, and subfractions S1–S6.

To further characterize the activity-associated constituents identified through spectrum–effect relationship analysis and activity validation, six representative constituents were quantitatively determined across the 95% EtOH and n-BuOH fractions, as well as sub-fractions S1–S6 (Figure 9). The results showed that CGA and 4-CQA were significantly enriched in Fr. n-BuOH compared with Fr. 95% EtOH and were mainly distributed in S1–S3. This distribution pattern was consistent with the observation that the overall activity of S1–S4 was superior to that of S5–S6. It also agreed with the conclusion from the spectrum–effect analysis that P1 (CGA) and P2 (4-CQA) made greater contributions, further supporting their roles as the key active constituents responsible for the cardioprotective effects of *A. venetum* leaves. In contrast, Myr-3-Gal and Myr-3-Glc were primarily distributed in S3–S5; combined with the subsequent synergistic effect results, this suggests that they are more likely to contribute to the overall effect as auxiliary or synergy-related constituents.

Notably, hyperoside and isoquercitrin, the officially recognized quality markers for *A. venetum* leaves in the current pharmacopoeia, were mainly enriched in the later fractions (S4–S6), with the highest contents found in S5 and S6. This result indicates that the officially recognized markers cannot adequately reflect the distribution characteristics of cardioprotective constituents in *A. venetum* leaves. Taking into account the quantitative distribution, spectrum–effect relationship, and activity validation results, CGA and 4-CQA can serve as candidate active constituents for the cardioprotection-oriented quality evaluation of *A. venetum* leaves, while S1–S3 can serve as the optimal fractions for the subsequent development of functional extracts.

## 3. Discussion

In this study, spectrum–effect analysis, zebrafish myocardial injury models, transcriptomics, and molecular docking were used to explore the cardioprotective mechanisms of *A. venetum* leaf subfractions. Spectrum–effect analysis (GRA and OPLS), combined with the results from the ISO-induced zebrafish myocardial injury model, suggested that CGA was a major activity-associated candidate compound closely associated with pharmacological activity. The cardioprotective effects of CGA, previously noted in ISO-induced injury models, involve energy metabolism enhancement, stabilization of cardiac mitochondrial enzyme activities, AMPK/SIRT1 and Wnt/β-catenin pathway regulation [24,25,26,27]. In the present study, the protective effect of CGA was further validated in a zebrafish myocardial injury model, providing zebrafish-based in vivo evidence for its contribution to the activity of *A. venetum* leaves. Additionally, the combination of CGA and Myr-3-Glc (Group C) in H9c2 cells enhanced cardioprotection compared to CGA alone. Mechanistic studies revealed that Group C reduced myocardial inflammation and apoptosis by enhancing PPARα signaling (Figure 8), which is crucial for energy metabolism [18] and cardiomyocyte health [17]. Moderate restoration or activation of PPARα provides cardioprotective effects, whereas its inhibition leads to apoptosis and heart dysfunction [20,21,22]. Previous research has indicated that PPARα may play a role in cardioprotection by modulating processes including fatty acid metabolism, inflammatory responses, and mitochondrial function [22,23]. Nonetheless, as the current study did not directly evaluate mitochondrial function, additional research is necessary to ascertain whether mitochondrial regulation is implicated in the PPARα/RXRα-mediated cardioprotective effects of CGA and its active combinations. This study confirmed that PPARα inhibitors negate the protective effects of Group C, highlighting the importance of this pathway.

Previous research has demonstrated that extracts from *A. venetum* leaves exhibit a range of cardioprotective effects, such as the inhibition of myocardial fibrosis, a reduction in ischemia–reperfusion-induced oxidative stress, the mitigation of anthracycline-induced cardiotoxicity, and the modulation of intestinal metabolites [5,6,8,28,29,30]. However, the majority of studies have concentrated on crude extracts without systematically identifying active fractions and core constituents, thereby limiting the potential for the targeted development and application of active principles in traditional Chinese medicine. To address this limitation, the present study employed an activity-guided fractionation strategy. The 95% ethanol extract of *A. venetum* leaves was fractionated. Subsequent bioactivity screening revealed that the n-butanol fraction exhibited the most potent anti-myocardial injury activity, meriting further detailed investigation. By employing integrated spectrum–activity correlation and pharmacological validation, CGA was identified as the primary active component responsible for the bioactivity of this fraction. This strategy not only addresses the limitations of previous studies based on crude extracts but also offers an approach for the precise development of traditional Chinese medicine and the discovery of new bioactive fractions.

This study integrated spectrum–effect relationship analysis with VER- and ISO-induced zebrafish myocardial injury models to create an effective platform for compound screening and mechanistic investigation. Spectrum–effect analysis enables quantitative correlation between chemical constituents and pharmacological effects, offering objective evidence for identifying key bioactive compounds [31]. Zebrafish, characterized by high genetic conservation, optical transparency, and rapid development, are useful early-stage models for assessing cardiac function, cardiotoxicity, and screening efficacy in complex herbal systems [14,32,33]. Compared with traditional rodent models, zebrafish cardiac injury models offer shorter modeling cycles, stable drug exposure, and ease of handling, making them widely used for the early screening of active compounds to evaluate potential cardioprotective effects [34,35]. This study employed a VER-induced acute injury model for rapid preliminary screening and an ISO-induced chronic injury model for detailed mechanistic investigation [13,14,15]. The present study extends previous cardioprotective research on *A. venetum* leaves by integrating HPLC fingerprint profiling with zebrafish-based anti-myocardial injury evaluation to establish a spectrum–effect relationship for its small-molecule constituents. Moreover, by combining spectrum–effect analysis with zebrafish-based pharmacological evaluation, this study provides novel evidence for the application of zebrafish models in natural product screening, functional component identification, and preliminary cardioprotective mechanism exploration.

## 4. Materials and Methods

### 4.1. Establishment of Fingerprint Spectra for the n-BuOH Part of an Aqueous EtOH Extract of A. venetum Leaves

Plant materials: Dried leaf specimens of *A. venetum* (Apocynaceae) were procured from a location situated at approximately 37.2458° N latitude and 118.6068° E longitude. The botanical identity of the plant material was verified by Professor Fengqin Zhou from the School of Pharmacy at Shandong University of Traditional Chinese Medicine. A voucher specimen, designated as HYZY-2022-A.VL, has been deposited in the Herbarium of the Research Institute of Marine Traditional Chinese Medicine, Qingdao Academy of Chinese Medicine.

Sample preparation: The dried leaves of *A. venetum* were ground and passed through a 50-mesh sieve. Approximately 400 g of sample was weighed and extracted twice under reflux with 95% ethanol for 1 h each time, using solvent-to-sample ratios of 10:1 and 8:1 (*v*/*w*), respectively. The filtrates were combined and concentrated under reduced pressure to obtain the crude extract. The crude extract was re-dispersed in water and successively partitioned with ethyl acetate and n-butanol to obtain Fr. EtOAc, Fr. n-BuOH, and the remaining aqueous fraction (Fr. H_2_O) as intermediate fractions for subsequent separation. The Fr. n-BuOH fraction was loaded onto an HP_2_MGL macroporous adsorption resin column and sequentially eluted with aqueous ethanol (0%, 10%, 20%, 30%, 40%, 60%, 75%, and 95%, *v*/*v*). The resulting eight eluates (S1–S8) were collected, freeze-dried to yield powders, and stored for subsequent use.

HPLC chromatogram acquisition: A Waters 1525 HPLC system equipped with a Kromasil 100-5 C_18_ column (5 μm, 4.6 mm × 250 mm, Milford, MA, USA) was used. The column temperature was 40 °C. The detection wavelength was 256 nm. The mobile phase included acetonitrile (A) and 0.1% trifluoroacetic acid aqueous solution (B). The following gradient elution program was used: 0–12 min, 12% → 24% (A); 12–32 min, 24% → 40% (A). The injection volume was 10 μL. The flow rate was 1 mL/min. Chromatographic files S1–S6 were imported into the “Chinese Herbal Medicine Chromatographic Fingerprint Similarity Evaluation System (2012 Edition, Chinese Pharmacopoeia Commission)” to screen for common characteristic peaks. Peak 8 (isoquercitrin) was designated as the reference peak. The relative retention times and relative peak areas of each common peak were calculated for subsequent chemometric analysis. Methodological details regarding the materials and reagents are provided in the Supplementary Materials.

### 4.2. UPLC–QE–Orbitrap–MS/MS Analysis and Structural Identification

UPLC analysis method: A Waters Acquity UPLC BEH C_18_ column (1.7 µm, 2.1 mm × 100 mm) maintained at 40 °C was used. The mobile phase consisted of pure water (A) and methanol (B). The linear gradient elution program was as follows: 0–3 min, 5–35% B; 3–13 min, 35–60% B; 13–15 min, 60% B; 15–23 min, 60–88% B; 23–24 min, 88–100% B; 24–39 min, 100% B; 39–40 min, 100–5% B; 40–50 min, 5% B; injection volume: 2 µL; flow rate: 0.2 mL/min; and full wavelength scan: 190–700 nm.

Mass spectrometry method: The experiments were conducted on a Q Exactive Plus mass spectrometer (Thermo Fisher Scientific, Waltham, MA, USA) with a mass range of *m*/*z* 150–2000 Da for both positive and negative ion modes. The detailed mass spectrometry parameters were as follows: capillary voltage, 3.50 kV (ESI^+^) and 4.00 kV (ESI^−^); ion source temperature, 120 °C; sample cone voltage, 40 V; cone gas flow rate, 50 L/h; desolvation temperature, 500 °C; and desolvation gas flow rate, 800 L/h. Low collision energy was set at 6 eV, with high collision energy ranging from 10 to 45 eV. External calibration for mass accuracy was performed prior to analysis.

### 4.3. Quantitative Analysis of Six Representative Constituents

Quantitative analysis was performed for six representative constituents in Fr. 95% EtOH, Fr. n-BuOH, and subfractions S1–S6 of *A. venetum* leaves, including CGA, 4-CQA, Myr-3-Gal, Myr-3-Glc, hyperoside, and isoquercitrin. The chromatographic analysis was carried out on the same HPLC system under the same conditions as described in Section 2.1. Reference standards of the six constituents were accurately weighed and dissolved in methanol to prepare a series of mixed standard solutions at appropriate concentrations. Calibration curves were established by plotting peak area against concentration for each constituent. The contents of the six constituents in each sample were determined from the corresponding calibration curves and expressed as mg/g of sample.

### 4.4. Evaluation of Anti-Myocardial Injury in a Drug-Induced Zebrafish Model

The experiments utilized 4-month-old wild-type AB-strain zebrafish and 3-month-old Tg(*myl7:GFP*) heart-specific green fluorescent transgenic zebrafish, both of which were purchased from the National Zebrafish Resource Center and reared and bred in our laboratory. This study did not involve adult zebrafish and no invasive procedures were performed on larvae. All experimental procedures were performed in accordance with national and international guidelines (EU Directive 2010/63/EU). The zebrafish embryos used in this study were all younger than 5 days post-fertilization (dpf), a developmental stage at which sex cannot be phenotypically distinguished. The detailed rearing and breeding conditions are provided in the Supplementary Materials, Methods S2.

Establishment of an acute myocardial injury model: Following and optimizing the method described by Li et al., a VER-induced acute myocardial injury model was established in zebrafish [36]. Zebrafish embryos at 48 hpf (hours post-fertilization) were randomly distributed into 6-well plates, with 30 embryos per well. Prior to modeling, the embryos were preincubated for 4 h in solutions of different-polarity fractions of *A. venetum* leaves (Fr. S1–S6, 250 μg/mL) or the positive control digoxin (10 μM). Digoxin and the tested fractions were first dissolved in DMSO to prepare high-concentration stock solutions and then diluted with E3 medium to the required working concentrations before use. The final concentration of DMSO did not exceed 0.1% (*v*/*v*). The blank control group was maintained in E3 medium, and VER was prepared in E3 medium. VER (40 μM) was then added, and co-incubation was continued for 20 min. The treatment concentrations were determined on the basis of zebrafish survival assays, as described in the Supplementary Materials, Methods S3.

Establishment of a chronic myocardial injury model: Based on a modified method described previously [15] and optimized, a chronic myocardial injury model in zebrafish was induced using ISO. Normally developing Tg(*myl7:GFP*) embryos were distributed into 24-well plates at 24 hpf (10 embryos per well) and continuously exposed to ISO (1 mM) alone or in combination with the tested compounds until 72 hpf. ISO was prepared in E3 medium, whereas the tested compounds were first dissolved in DMSO to prepare high-concentration stock solutions and then diluted with E3 medium to the required working concentrations before treatment. The final concentration of DMSO did not exceed 0.1% (*v*/*v*). All dosages were determined using survival assays, as detailed in the Supplementary Materials, Methods S3.

Image-based quantitative phenotyping of zebrafish cardiac function: Whole-body morphology was captured under a stereomicroscope (50× magnification), and the areas of PA and VA were measured using ImageJ software (version 1.53a). HR was manually determined by recording the number of heartbeats within 15 s under the microscope and multiplying the value by 4 to obtain beats per minute. BFV and CO were measured using the MicroZebraLab ZEB6245 system. Using a stereoscopic fluorescence microscope (11.2× magnification), 20 consecutive images were captured, from which end-diastolic and end-systolic ventricular images were extracted. Image-Pro Plus software (version 6.0.0.260) with a selection tool was used to accurately delineate the long-axis diameter (DL), short-axis diameter (DS), end-diastolic short-axis diameter (Dd), and end-systolic short-axis diameter (Ds) for calculating the EF, SV, and FS. According to a previously reported image-based cardiac function assessment method in embryonic zebrafish, ventricular volume was estimated by assuming a prolate spheroid shape of the ventricle, and EF and FS were calculated from end-diastolic and end-systolic ventricular dimensions [37]. The formulas are as follows: V = 0.523 × DL × DS^2^; EDV = max(V); ESV = min(V); SV = EDV − ESV; EF = (EDV − ESV)/EDV × 100%; and FS = (Dd − Ds)/Dd. The effects of subfractions S1–S6 on myocardial injury were evaluated using the above qualitative and quantitative indicators.

### 4.5. Spectrum–Effect Relationship Analysis

Grey relational analysis (GRA) and orthogonal partial least squares analysis (OPLS) were employed to evaluate the spectrum–effect relationship between the HPLC fingerprint spectra and zebrafish myocardial injury protection evaluation indicators. The evaluation indicators included the PA, VA, CO, BFV, HR, FS, SV, and EF, which comprehensively reflected the protective efficacy against myocardial injury.

Following the method of Chen et al. [38], the improvement rate of each pharmacodynamic indicator was used as the reference sequence (*X_o_*), whereas the relative peak area of the common peaks was used as the comparison sequence (*X_i_*). All datasets were normalized using the equalization method to obtain dimensionless standardized sequences (*X_o_*′ and *X_i_*′). The absolute difference between the standardized reference sequence and comparison sequence was calculated using the following formula: $\Delta_{\mathrm{oi}}(k)=|X_{o}^{\prime }\left(k\right)\;-\;X_{i}^{\prime }\left(k\right)|$. A resolution coefficient of ρ = 0.5 was applied to balance the discrimination power and model stability. The grey relational coefficient was calculated according to the following formula: $\eta_{oi}(k)$ = $\frac{min\;min\;\Delta_{oi(k)}+\rho *max\;max\;\Delta_{oi(k)}}{\Delta_{oi(k)}+\rho \;max\;max\;\Delta_{oi(k)}}$. The correlation degree (R), defined as the mean value of all grey relational coefficients, was calculated as follows: $r_{oi}$ = $\frac{1}{n}\sum_{k=1}^{n}\eta_{oi(k)}$.

OPLS was established using SIMCA 14.1 software, following a previously described method [39], with the peak area of common peaks as the independent variable (*X*) and the improvement rate of each biological indicator as the dependent variable (*Y*). The absolute values of the regression correlation coefficients were used to estimate the contribution of each peak to biological efficacy, and VIP scores were applied to screen peaks strongly associated with anti-myocardial injury activity.

### 4.6. Transcriptome Sequencing and qRT–PCR Analysis

RNA-seq technology was used to analyze transcriptome changes in zebrafish across the control, ISO, and CGA groups [40], with six biological replicates per group. Following quality control using the Agilent 2100 Bioanalyzer (Agilent Technologies, Santa Clara, CA, USA) library preparation and sequencing were performed on the Illumina NovaSeq 6000 PE150 platform (Illumina, San Diego, CA, USA). The reference genome used was ensembl_109_danio_rerio_grcz11_primary. Differentially expressed genes (DEGs) between groups were analyzed using the DESeq2 R package, with screening criteria set at corrected *p* < 0.05 and |log2(fold change)| > 1. The functional annotation of the identified DEGs was performed using clusterProfiler, including GO enrichment analysis and KEGG pathway analysis.

Quantitative reverse transcription polymerase chain reaction (qRT–PCR) was performed using a qTower^3^G real-time fluorescent quantitative PCR instrument (Analytik, Jena, Germany) to validate the expression levels of the DEGs identified in the RNA-seq results. Total RNA from zebrafish was extracted using TRIzol reagent. The primers used were designed using Oligo 7 software (sequences listed in the Supplementary Materials, Table S1). The SPARKscript II RT Plus Kit (with gDNAEraser) and 2 × SYBR Green qPCR Mix Kit (Shandong Sparkjade Biotechnology Co., Ltd., Jinan, China) were used for reverse transcription and qRT–PCR analysis of the DEGs. PCR protocols followed previously established methods with minor modifications [41]. The relative expression levels of selected genes were analyzed using the 2^−ΔΔCT^ method.

### 4.7. Molecular Docking

To further investigate the interaction between CGA and key target proteins, molecular docking analysis was conducted. The 2D structure of the CGA was downloaded from PubChem (https://pubchem.ncbi.nlm.nih.gov/, accessed on 28 February 2025) on the basis of its CAS number. The receptor proteins pparaa (UniProt: Q5RGZ2), pparda (UniProt: F8W3D2), and pparg (UniProt: P37231) were selected, and their 3D structures were obtained from the UniProt database (https://www.uniprot.org/, accessed on 28 February 2025). After dehydration, ligand removal, and hydrogenation pretreatment of the proteins, docking analysis was performed using the CDOCKER algorithm within the receptor–ligand interaction module of Discovery Studio 2019 R2 software. Docking parameters were set as follows: Pose Cluster Radius 0.5, Random Conformations 10, Orientations to Refine 10, and all other settings default. The binding stability between ligands and receptors was evaluated using binding energy values, where a lower energy indicates more stable binding and stronger affinity.

### 4.8. Evaluation of Anti-Myocardial Injury in ISO-Induced H9c2 Cells

The H9c2 cell line was purchased from Procell (Wuhan, China) and cultured in H9c2 (2-1) specific medium (Procell, Wuhan, China) at 37 °C under 5% CO_2_ conditions. Cells were seeded into 96-well plates and cultured for 24 h before treatment with varying concentrations of CGA, 4-CQA, Myr-3-Gal, and Myr-3-Glc for 48 h. The control group was treated with H9c2 (2-1) specific medium. The active compounds were prepared as DMSO stock solutions and diluted with H9c2 (2-1) specific medium before use, with a final DMSO concentration not exceeding 0.1% (*v*/*v*). Cell viability was assessed using the MTT assay. In the ISO-induced injury assay, cells were exposed to ISO (135 μM) alone or together with different concentrations of the active compounds. Synergy scores were calculated using SynergyFinder+ from the experimentally measured dose–response matrices. As this study used matrix-based combination data with multiple concentration levels for each drug, the ZIP model was considered suitable for the primary analysis.

The experimental groups for the ISO-induced H9c2 cell injury model were as follows: (1) control group; (2) model group (135 μM ISO); (3) Group C-treated group (25 μM CGA + 6.25 μM Myr-3-Glc + 135 μM ISO); (4) PPARα inhibitor group (135 μM ISO + 10 μM GW6471); and (5) combined intervention group (25 μM CGA + 6.25 μM Myr-3-Glc + 135 μM ISO + 10 μM GW6471). Total protein from H9c2 cells was extracted using RIPA lysis buffer supplemented with protease and phosphatase inhibitors. Equal amounts of protein (30 μg) were separated by 10% SDS–PAGE and transferred to PVDF membranes. After blocking with 5% skim milk for 2 h, the following primary antibodies were added and incubated overnight at 4 °C: PPARα (66826-1-Ig; Proteintech (Rosemont, IL, USA), 1:1000), RXRα (21218-1-AP; Proteintech, 1:1000), Bcl-2 (D17C4; Cell Signaling (Danvers, MA, USA), 1:1000), Bax (A0207; ABclonal (Woburn, MA, USA), 1:1000), and β-actin (66009-1-Ig; Proteintech, 1:2000). The membranes were subsequently incubated at room temperature for 1 h with the following HRP-labeled secondary antibodies: goat anti-rabbit IgG-HRP (RGAR001; Proteintech, 1:4000) or goat anti-mouse IgG-HRP (RGAM001; Proteintech, 1:4000). The protein bands were detected and quantified using a Tanon 5200 fully automated chemiluminescence imaging system (Shanghai, China). Inflammatory cytokine levels were measured using ELISAs. H9c2 cell culture supernatants were collected, and IL-6, IL-1β, and TNF-α levels were determined according to the instructions of the kit (Shanghai Jianglai Biotechnology Co., Ltd., Shanghai, China). TUNEL staining was conducted using the One-step TUNEL In SituApoptosis Assay Kit (Elabscience Biotechnology Co., Ltd., Wuhan, China) following the manufacturer’s protocol. Nuclei were counterstained with DAPI using ProLong Gold antifade reagent (Invitrogen, Carlsbad, CA, USA).

### 4.9. Statistical Analysis

Statistical analysis was performed using GraphPad Prism 9.0 software (GraphPad Software, San Diego, CA, USA). The results are expressed as the mean ± standard deviation. Before statistical analysis, the normality of data distribution was assessed using the Shapiro–Wilk test, and homogeneity of variance was evaluated using the Brown–Forsythe test or Bartlett’s test, where appropriate. Comparisons between two groups were conducted using unpaired two-tailed Student’s *t* tests. Comparisons among multiple groups were performed using ordinary one-way analysis of variance (ANOVA), followed by Tukey’s multiple comparisons test for post hoc pairwise comparisons where appropriate. Adjusted *p* values from the corresponding post hoc tests were used to determine statistical significance. Where applicable, 95% confidence intervals and effect sizes were reported, with Cohen’s d for two-group comparisons and η^2^ for one-way ANOVA. Exact sample sizes for each experiment are provided in the corresponding figure legends. Statistical significance levels were set as follows: compared with the blank control group, ^##^ *p* < 0.01; compared with the model group, * *p* < 0.05, ** *p* < 0.01.

## 5. Conclusions

In this study, an activity-guided separation strategy was integrated with spectrum–effect relationship analysis and zebrafish-based pharmacological evaluation to identify candidate cardioprotective compounds from *A. venetum* leaves. CGA, a phenolic acid abundant in *A. venetum* leaves, was identified as a major activity-associated candidate compound based on its superior correlation and efficacy in both the spectrum–effect analysis and the ISO-induced zebrafish myocardial injury model. Notably, CGA combined with Myr-3-Glc (Group C) produced a previously unreported synergistic enhancement in cardioprotective activity in H9c2 cells. Mechanistic studies further revealed that this combination mitigated myocardial inflammation and apoptosis by activating PPARα signaling. Quantitative analyses revealed that CGA and 4-CQA were primarily concentrated in fractions S1–S3, indicating that these subfractions may contribute substantially to the cardioprotective potential of *A. venetum* leaves. Overall, this integrated strategy helped clarify the active basis of *A. venetum* leaves and highlighted the value of zebrafish models in the early-stage pharmacological evaluation of natural products.

Despite these findings, certain limitations remain. Although zebrafish and H9c2 cell models are valuable for preliminary screening and mechanistic validation, they do not fully recapitulate mammalian cardiac physiology, myocardial injury, or responses of mature cardiomyocytes. Accordingly, the present findings should be interpreted as early-stage pharmacological evidence rather than direct evidence of therapeutic efficacy in mammals. Additionally, the study assessed the activity-associated distribution of CGA and 4-CQA only in the n-butanol fraction and its subfractions, whereas other fractions, such as the ethyl acetate fraction, were not further investigated. Further research utilizing mammalian models is required to assess the bioavailability and pharmacokinetics of CGA and 4-CQA, as well as to compare various fractions and evaluate mitochondrial respiratory function, membrane potential, and ATP production. This will help elucidate the pharmacological significance and translational potential of these compounds derived from A. venetum leaves. Additionally, it is essential to investigate whether the PPARα/RXRα signaling pathway plays a role in the regulation of mitochondrial function by CGA and its active combinations.

## Figures and Tables

**Table 1:** Identification information for the 12 characteristic chromatographic peaks.

Peak No.	Retention Time (min)	[M − H]− (*m*/*z*)	Formula	Error (ppm)	MS/MS (*m*/*z*)	Identification
1	5.79	353.0831	C_16_H_18_O_9_	−2.2235	191.0561, 179.0350, 173.0456, 135.0451	Chlorogenic acid
2	5.95	353.0880	C_16_H_18_O_9_	−2.0507	191.0561, 173.0457, 161.0242, 135.0451	4-caffeoylquinic acid
3	10.70	479.0831	C_21_H_20_O_13_	−1.4010	316.0225, 287.0198, 271.0250,	Myricetin 3-*O*-galactoside
4	10.98	479.0833	C_21_H_20_O_13_	−1.7832	316.0224, 287.0200, 271.0246	Myricetin 3-*O*-glucoside
5	12.06	567.2087	-	−0.8911	431.1923, 393.1770, 307.1399	Unidentified
6	12.47	439.1822	-	2.7150	429.1536, 389.1822, 393.1770,	Unidentified
7	13.14	463.0883	C_21_H_20_O_12_	−1.6233	300.0276, 271.0249, 255.0300, 243.0296,	Hyperoside
8	13.49	463.0882	C_21_H_20_O_12_	−1.8210	300.0276, 271.0249, 255.0300, 243.0300	Isoquercitrin
9	14.41	549.0886	C_24_H_22_O_15_	2.0616	505.0988, 301.0353, 300.0275, 271.0249, 151.0034	Quercetin-3-*O*-(6″-*O*-malonyl)-galactoside
10	15.05	447.0933	C_21_H_20_O_11_	−1.6566	415.1975, 255.0293, 227.0345	Kaempferol-3-*O*-galactoside
11	15.84	431.0984	C_21_H_20_O_10_	1.3439	269.0455, 268.0377, 240.0423	Apigenin 7-*O*-glucoside
12	16.09	505.0989	C_23_H_22_O_13_	−1.1972	489.1040, 301.0353, 271.0249, 255.0300, 151.0036	Quercetin 3-*O*-(6″-*O*-acetyl)-glucoside

## Data Availability

The original contributions presented in this study are included in the article/Supplementary Materials. Further inquiries can be directed to the corresponding authors.

## Supplementary Materials

The following supporting information can be downloaded at: https://www.mdpi.com/article/10.3390/ph19060879/s1, Methods S1. Materials and reagents; Methods S2. The breeding conditions and ethical considerations pertaining to zebrafish; Methods S3. Dose safety evaluation; Results S1. Result of methodology verification; Results S2. Total ion chromatograms and MS/MS fragmentation data of Fr. n-BuOH; Results S3. Differential gene expression and GO enrichment analysis; Result S4 Evaluation of four candidate compounds against myocardial injury in H9c2 cells; Table S1: Sequences of primers used for qRT–PCR; Table S2: Result of method validation for HPLC fingerprint; Table S3. Model parameters of OPLS analyses for eight myocardial injury-related indicators; Figure S1. Effects of S1–S6 subfractions on zebrafish embryo survival and morphology (n = 30); Figure S2. PDA/TIC chromatograms of Fr. n-BuOH and MS/MS fragmentation patterns with proposed cleavage pathways of peaks 1–4; Figure S3. Differential gene expression and GO enrichment analysis; Figure S4. Protective effects of four candidate compounds against ISO-induced injury in H9c2 cells.

## Abbreviations

The following abbreviations are used in this manuscript:

4-CQA	4-caffeoylquinic acid
BFV	Blood flow velocity
CGA	Chlorogenic acid
CO	Cardiac output
Dd	End-diastolic short-axis diameter
DEGs	Differentially expressed genes
DL	Long-axis diameter
DS	Short-axis diameter
Ds	End-systolic short-axis diameter
EDV	End-diastolic ventricular volume
EF	Ejection fraction
ESV	End-diastolic ventricular volume
FS	Fractional shortening
GRA	Grey relational analysis
hpf	Hours post-fertilization
HPLC	High-performance liquid chromatography
HR	Heart rate
ISO	Isoprenaline
Myr-3-Gal	Myricetin 3-*O*-galactoside
Myr-3-Glc	Myricetin 3-*O*-glucoside
OPLS	Orthogonal partial least squares analysis
PA	Pericardial edema area
qRT–PCR	Quantitative reverse transcription polymerase chain reaction
SV	Stroke volume
VA	Venous congestion area
VER	Verapamil
