# Supplementary material for: Exploring the Cardioprotective Spectrum–Effect Relationship of Apocynum venetum L. Using a Zebrafish Model
Source: Pharmaceuticals (Basel). 2026 May 31;19(6):879. doi: 10.3390/ph19060879 (PMC13305498; doi:10.3390/ph19060879)
Supplement: Supplementary file 1 [file pharmaceuticals-19-00879-s001.zip › pharmaceuticals-4331155-supplementary.pdf]

Supplementary Materials

Methods S1. Materials and reagents

HP2MGL macroporous adsorption resin was purchased from GreenHerbs Science and Technology Co., Ltd. (Beijing, China). Verapamil hydrochloride and methylcellulose were purchased from Aladdin Biochemical Technology Co., Ltd. (Shanghai, China). Digoxin was purchased from TCI Development Co., Ltd. (Shanghai, China). Chlorogenic acid, 4-caffeoylquinic acid, myricetin 3-*O*-galactoside, and myricetin 3-*O*-glucoside (purity ≥98%) were purchased from Desite Biological Technology Co., Ltd. (Chengdu, China). Isoproterenol hydrochloride was purchased from Shanghai yuanye Bio-Technology Co., Ltd. (Shanghai, China). SPARKscript II RT Plus Kit (With gDNAEraser), and 2 × SYBR Green qPCR Mix Kit were purchased from Shandong Sparkjade Biotechnology Co., Ltd. (Jinan, China). DMSO, 95% ethanol, calcium chloride dihydrate, magnesium sulfate heptahydrate, potassium chloride, and sodium chloride were purchased from Sinopharm Chemical Reagent Co., Ltd. (Shanghai, China). Zebrafish embryos were maintained in E3 medium containing 5 mM NaCl, 0.17 mM KCl, 0.33 mM $CaCl_2$, and 0.33 mM $MgSO_4$. Methanol and acetonitrile were purchased from Anhui Fulltime Specialized Solvent & Reagent Co., Ltd. (Anqing, China).

Methods S2. The breeding conditions and ethical considerations pertaining to zebrafish

Adult zebrafish were maintained under a consistent 14-hour light and 10-hour dark cycle photoperiod at a temperature of 28 ± 0.5 °C within an automated zebrafish housing system (Jinshuihaiyang, Qingdao, China). The fish were provided with live brine shrimp as their diet twice daily. Male and female zebrafish were bred at a 1:2 ratio to obtain fertilized eggs. Embryo water supplemented with 1 mg/L methylene blue was used for egg storage. All experiments complied with national and international guidelines (EU Directive 2010/63/EU). The zebrafish embryos used in this experiment were all younger than 5 days postfertilization (dpf), and the embryos at this stage are not distinguishable between males and females.

Methods S3. Dose safety evaluation

Prior to model establishment, a dose safety assessment was conducted for the *A. venetum* subfractions S1–S6 and four active compounds (peaks 1–4). Zebrafish embryos at 48 hours post-fertilization (hpf) were exposed to S1–S6 subfractions at concentrations of 62.5, 125, 250, 500, and 1000 μg/mL, and morphological changes and mortality were monitored up to 72 hpf. Normal morphology and no mortality were observed at concentrations ≤250 μg/mL; therefore, 250 μg/mL was selected as the experimental concentration (Figure S1). Similarly, the survival rates of embryos were evaluated after exposure to chlorogenic acid, 4-caffeoylquinic acid, myricetin 3-*O*-galactoside, and myricetin 3-*O*-glucoside at 50, 100, 200, 400 and 600 μM for 48, 72, and 96 hpf. No zebrafish mortality was observed at concentrations of 200 μM and below for each compound. Consequently, a concentration of 250 μg/mL was used for the *A. venetum* subfractions in the acute myocardial injury model, and 200 μM was used for the compounds in the chronic myocardial injury model.

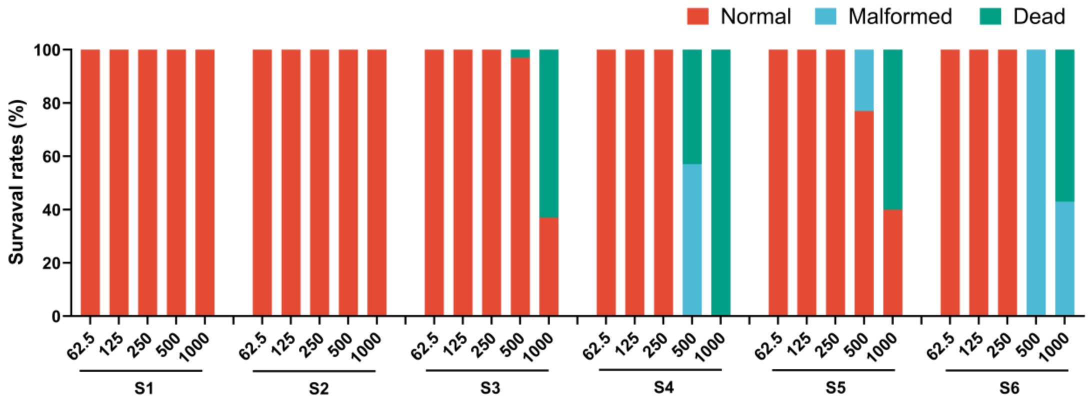

Figure S1. Effects of S1–S6 subfractions on zebrafish embryo survival and morphology (n = 30).

Table S1. Sequences of primers used for qRT–PCR

Gene	Forward Primer Sequences (5′-3′)	Reverse Primer Sequences (5′-3′)
β-actin	AGAGCTATGAGCTGCCTGACG	CCGCAAGATTCCATACCCA
pparaa	GCCAGTGTAACGAATAGCCTCA	ATTGCACTTGTCTAGCTTACTCC
pparda	ACTGACTGCCTGTCCAATCAACC	GCACAACGCTCCATTGCAGA
pparg	CCATCCTGTCCGGGAAGACCA	ATCATCTGCTCGCCTTCCACCA
cd36	CCCCAACTTAGATGAACACTCC	GGCCGTACATCATGTTGACC
rxraa	ATGCCATCAAATTCACCCAACGA	GAATCCTCTTCGCCCACTCCAC

Results S1. Result of methodology verification

Method validation results demonstrated that the analytical method exhibited excellent precision, stability, and repeatability. Specifically, in the precision test, the RSD values of the relative retention times for all common peaks were below 0.11%, and the RSD values of the relative peak areas were below 3.84%, indicating high measurement accuracy. In the stability test, the RSD values of retention times and peak areas were below 0.22% and 2.79%, respectively, confirming that the sample solution remained stable within 24 h. The repeatability test further verified the reliability of the method, with RSD values of relative retention times and relative peak areas for all common peaks below 0.07% and 2.65%, respectively. The detailed results are summarized in Table S2.

Table S2. Result of method validation for HPLC fingerprint

Common peaks	RSD(%)					
	Precision test		Stability test		Repetitive test	
	Retention time	Peak area	Retention time	Peak area	Retention time	Peak area
Peak 1	0.11	0.28	0.22	0.34	0.06	0.73
Peak 2	0.04	3.84	0.17	2.79	0.07	1.39
Peak 3	0.07	2.66	0.11	2.38	0.07	2.65
Peak 4	0.02	1.22	0.09	1.20	0.07	1.03

Peak 5	0.05	2.28	0.07	2.05	0.05	1.81
Peak 6	0.02	1.62	0.03	1.10	0.03	1.69
Peak 7	0.03	0.36	0.03	0.38	0.02	0.46
Peak 8	0.00	0.00	0.00	0.00	0.00	0.00
Peak 9	0.03	0.60	0.05	0.50	0.02	1.35
Peak 10	0.03	0.48	0.08	0.45	0.03	0.66
Peak 11	0.04	0.62	0.11	0.50	0.03	1.23
Peak 12	0.03	1.78	0.09	1.55	0.03	2.18

Results S2. Total ion chromatograms and MS/MS fragmentation data of Fr. n-BuOH

To supplement the chromatographic and structural information presented in the main text, the total ion chromatograms (TICs) of the n-butanol fraction of *A. venetum* leaves in both positive and negative ion modes are shown in Figure S2A. Additionally, the MS/MS spectra and detailed fragmentation characteristics of P1 (chlorogenic acid), P2 (4-caffeoylquinic acid), P3 (myricetin 3-*O*-galactoside) and P4 (myricetin 3-*O*-glucoside) are presented in Figure S2B-E. These peaks were not included in the main figures; however, their inclusion here ensures completeness and transparency in the compound identification process.

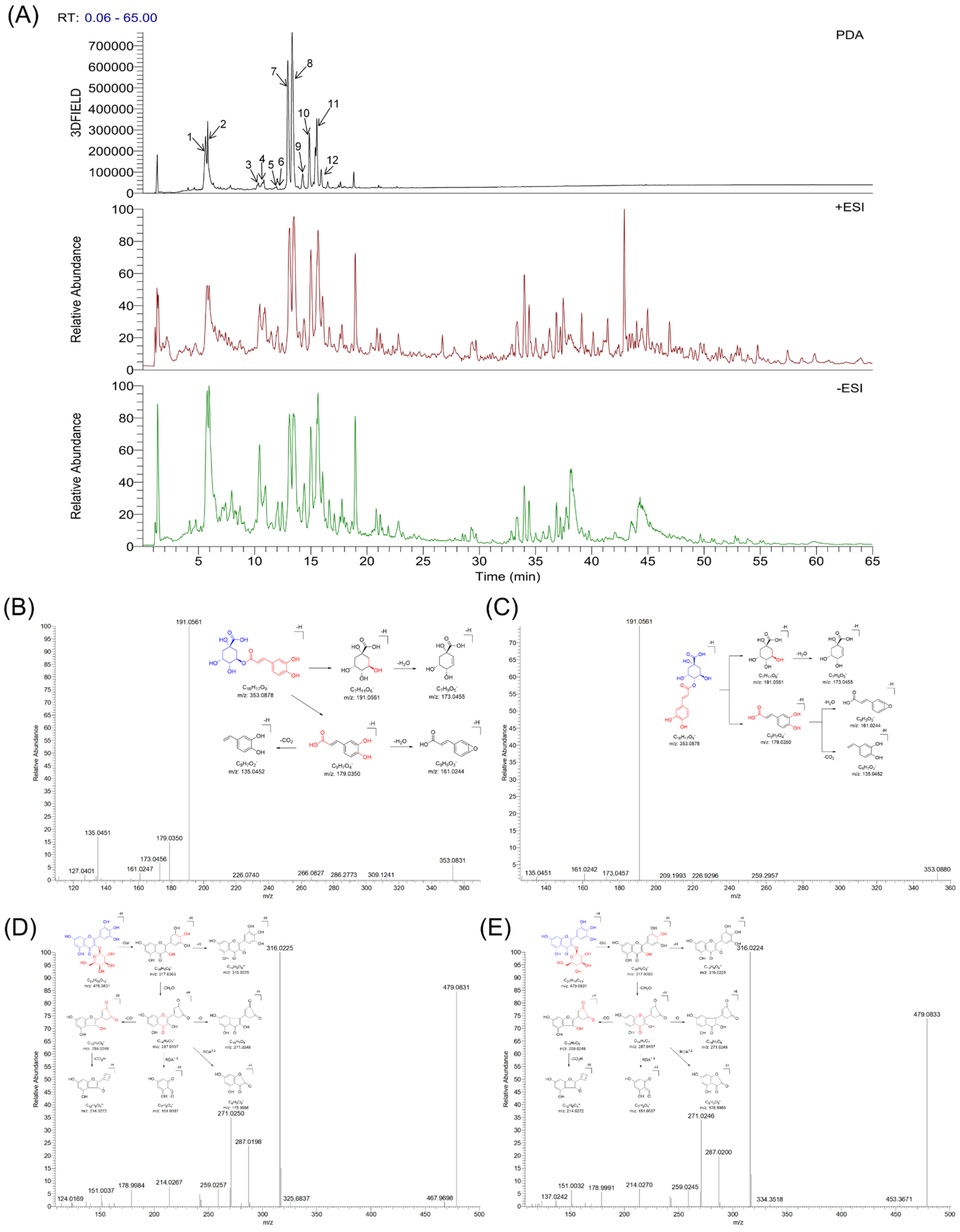

Figure S2. PDA/TIC chromatograms of Fr. n-BuOH and MS/MS fragmentation patterns with proposed cleavage pathways of Peaks 1–4. **(A)** PDA chromatogram and total ion chromatograms (TICs) acquired in the positive- and negative-ion modes, **(B)** chlorogenic acid, **(C)** 4-caffeoylquinic acid, **(D)** myricetin 3-*O*-galactoside, and **(E)** myricetin 3-*O*-glucoside.

Table S3. Model parameters of OPLS analyses for eight myocardial injury-related indicators

Indicator	R^2 (cum)	Q^2 (cum)
Pericardial edema area	0.980	0.951
Venous congestion area	0.917	0.839
Cardiac output	0.804	0.641
Blood flow velocity	0.878	0.707
Heart rate	0.989	0.976
Fractional shortening	0.968	0.908
Stroke volume	0.998	0.996
Ejection fraction	0.993	0.979

Results S3. Differential gene expression and GO enrichment analysis

Differential gene expression analysis was performed using the DESeq algorithm, with screening criteria set at $|\log_2(\text{fold change})| > 1$ and $P < 0.05$. Volcano plots were generated for visualization （Figure S3A-B）, where each dot represents a differentially expressed gene: red indicates significantly upregulated genes, green indicates significantly downregulated genes, and blue represents genes without significant changes.

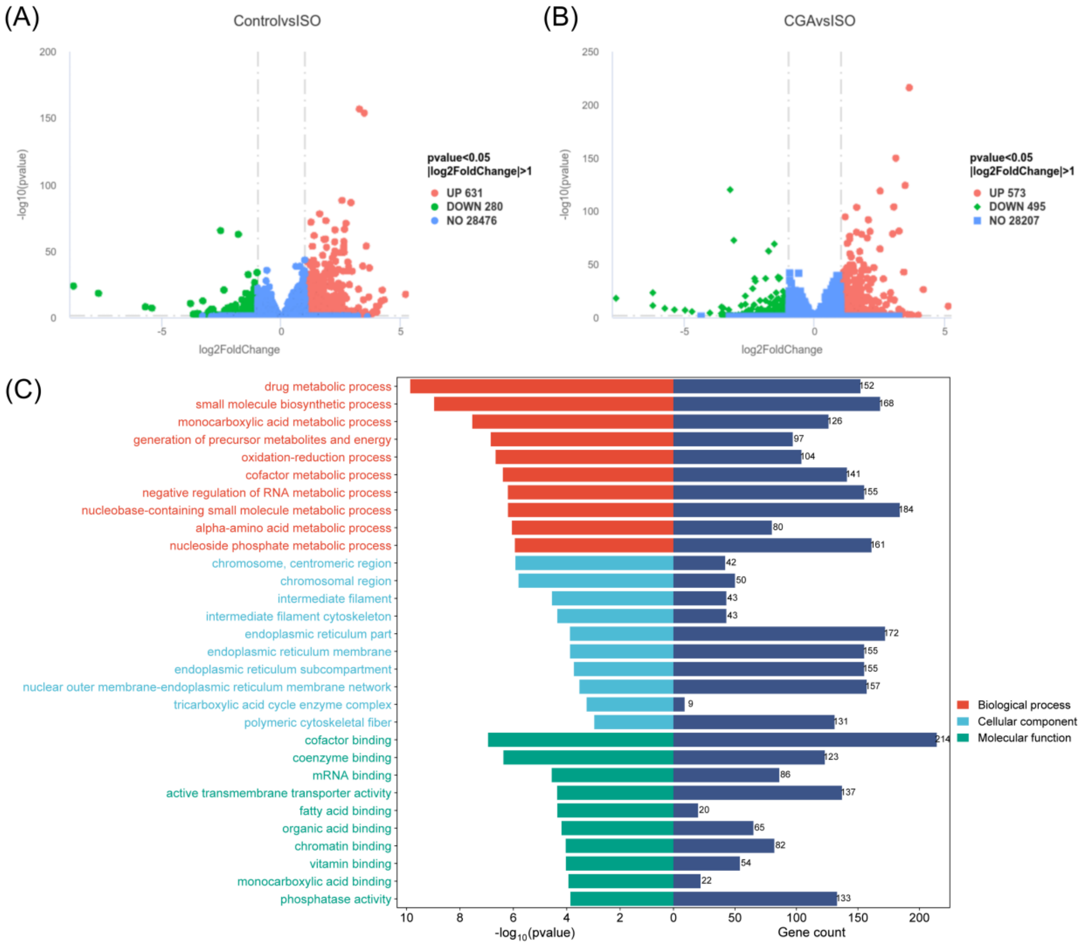

Figure S3. Differential gene expression and GO enrichment analysis. (A–B) Volcano plots of differentially expressed genes (A: Control vs ISO; B: CGA vs ISO). (C) GO enrichment analysis of the identified DEGs.

Result S4 Evaluation of four candidate compounds against myocardial injury in H9c2 cells

In the ISO-induced H9c2 cardiomyocyte injury model, all four candidate compounds exhibited protective effects to varying degrees, with differences in both strength and effective concentration ranges (Figure S4). Overall, CGA demonstrated the most pronounced and concentration-dependent protection, significantly enhancing cell viability at 3.125 μM and 6.25 μM ($P < 0.05$) and showing stronger effects across the 12.5–100 μM range ($P < 0.01$). 4-CQA and Myr-3-Gal provided moderate protection at 12.5–50 μM, whereas Myr-3-Glc exhibited relatively weak activity with a limited effective range, significantly improving cell viability only at 12.5 μM and 25 μM ($P < 0.05$). Cell viability was assessed using the MTT assay, and data are presented as mean ± SD (n = 3).

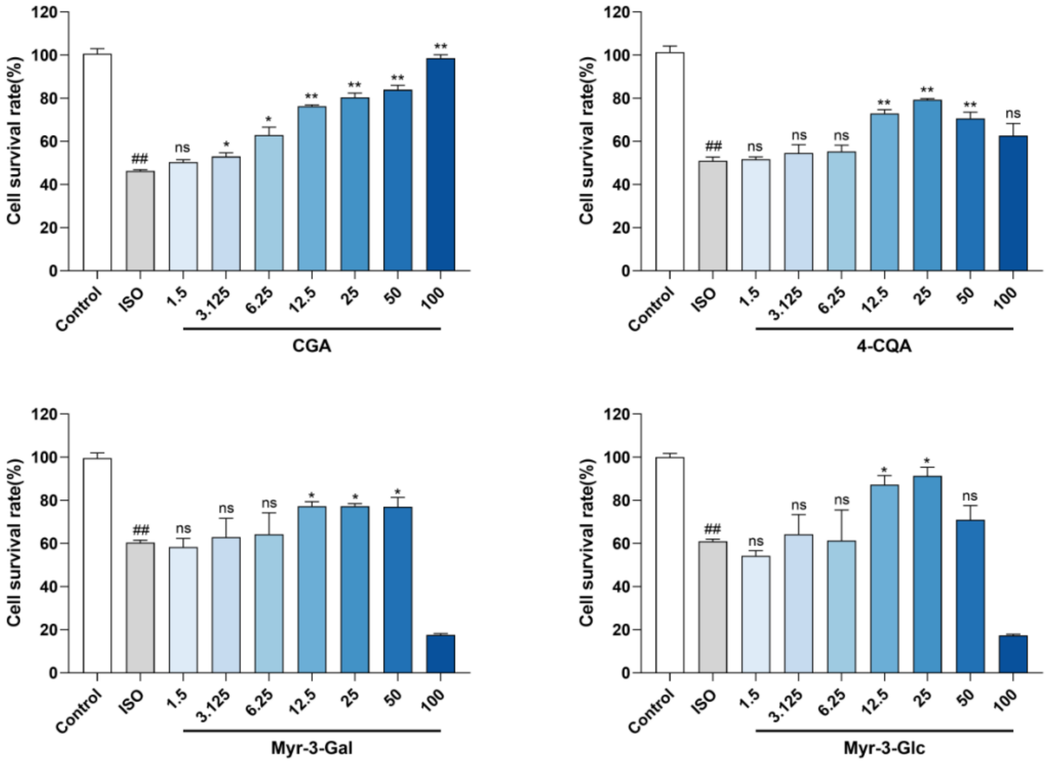

Figure S4. Protective effects of four candidate compounds against ISO-induced injury in H9c2 cells.
